# NEXT STEPS: ADVANCING CLINICAL TRIALS RESEARCH IN NURSING HOMES

**DOI:** 10.1093/geroni/igae098.4378

**Published:** 2024-12-31

**Authors:** Kathleen Unroe, Susan Hickman, Cari Levy, Sheryl Zimmerman, Alex Floyd, Debra Saliba

**Affiliations:** Indiana University, Indianapolis, Indiana, United States; Regenstrief Institute, Inc., Indianapolis, Indiana, United States; University of Colorado School of Medicine, Aurora, Colorado, United States; University of North Carolina at Chapel Hill, Chapel Hill, North Carolina, United States; Regenstrief Institute, Inc., Indianapolis, Indiana, United States; University of California Los Angeles, Los Angeles, California, United States

## Abstract

The National Institute on Aging recently funded the $15.6 million NEXT STEPs (Nursing home EXplanatory clinical Trials: Supporting Transformation by Enhancing Partnerships) initiative to create the organizational structure, processes, and procedures to develop a sustainable, diverse network of nursing home (NH) partners and researchers conducting clinical trials to guide optimal care. The perspectives and participation of interdisciplinary clinical providers and researchers are critical to inform the launch and activities of this innovative network. In addition to leveraging existing infrastructure and collaborations, NEXT STEPs embraces a comprehensive perspective of NH research that addresses six key elements of successful trials: 1) Designing the intervention, including anticipation of the study’s potential burden on NH staff and residents, and potential regulatory issues; 2) Ensuring equity throughout the trial’s design, conduct, analyses and dissemination; 3) Recruiting both NHs and individual participants, such as assessing and communicating the value of a given intervention to residents, providers, staff or leadership; 4) Cultivating engagement throughout the project, including plans to monitor and respond to internal or external events that affect projects; 5) Assessing outcomes, including identifying metrics and reliable data sources that can be used across projects; and 6) Following up and following through on communicating results and developing sustainable paths to implement successful interventions. Activities of this project will be guided through deep involvement with partner workgroups. This presentation will describe the evidence that supported the key elements and highlight the opportunities available for researchers, providers, and advocates to participate in the NEXT STEPS initiative.

